# Reactive Oxygen Species Are Involved in the Development of Gastric Cancer and Gastric Cancer-Related Depression through ABL1-Mediated Inflammation Signaling Pathway

**DOI:** 10.1155/2019/5813985

**Published:** 2019-07-15

**Authors:** Tianhe Huang, Fuling Zhou, Xiaohan Yuan, Tian Yang, Xuan Liang, Yu Wang, Honglei Tu, Jiantong Chang, Kejun Nan, Yongchang Wei

**Affiliations:** ^1^Department of Oncology, The First Affiliated Hospital of Xi'an Jiaotong University, Xi'an, 710061 Shaanxi Province, China; ^2^Department of Hematology, Zhongnan Hospital of Wuhan University, 430071 Hubei, China; ^3^Department of Oncology, The First Affiliated Hospital of Xinxiang Medical University, Weihui, 453100 Henan Province, China; ^4^Department of Radiation and Medical Oncology, Zhongnan Hospital of Wuhan University, Wuhan, 430071 Hubei, China; ^5^Department of General Surgery, The Second Affiliated Hospital of Xi'an Jiaotong University, Xi'an, 710004 Shaanxi Province, China

## Abstract

**Background:**

The mechanisms of crosstalk between depression and gastric cancer (GC) remain ill defined. Given that reactive oxygen species (ROS) is involved in the pathophysiology of both GC and depression, we try to explore the activities of ROS in the development of GC and GC-related depression.

**Methods:**

110 patients with newly diagnosed GC were recruited in our study. The clinical characteristics of these patients were recorded. Inflammation and oxidative stress markers were detected by ELISA. The depression status of patients with GC was assessed during follow-up. The association between ROS, ABL1, and inflammation factors was evaluated in H_2_O_2_-treated GC cell lines and The Cancer Genome Atlas (TCGA) database. The effect of ABL1 on inflammation was detected with Imatinib/Nilotinib-treated GC cell lines. A chronic mild stress- (CMS-) induced patient-derived xenograft (PDX) mice model was established to assess the crosstalk between depression and GC.

**Results:**

Depression was correlated with poor prognosis of patients with GC. GC patients with depression were under a high level of oxidative status as well as dysregulated inflammation. In the CMS-induced GC PDX mice model, CMS could facilitate the development of GC. Additionally, tumor bearing could induce depressive-like behaviors of mice. With the treatment of ROS, the activities of ABL1 and inflammatory signaling were enhanced both in vitro and in vivo, and blocking the activities of ABL1 inhibited inflammatory signaling.

**Conclusions:**

ROS-activated ABL1 mediates inflammation through regulating NF-*κ*B1 and STAT3, which subsequently leads to the development of GC and GC-related depression.

## 1. Background

Patients with gastric cancer (GC) are susceptible to mood disorder, especially to depression. We demonstrated that about 50% of investigated patients with GC suffered from depression and experienced poor quality of life [1]. Unfortunately, the unequivocal mechanism leading to the occurrence of depression for patients with GC remains poorly understood. We found that depression was associated with an impairment of immunologic functions and oxidative imbalance [2]. Oxidative stress (OS), characterized as excessive production of reactive oxygen species (ROS) [3], is involved in the initiation and development of cancer and depression. ROS lead to damage to tissues by attacking the DNA or disrupting cellular signaling [4, 5]. As a marker of OS, 8-hydroxy-2′-deoxyguanosine (8-OHdG) is dramatically elevated in serum samples of patients with GC. Furthermore, we have shown that levels of ROS are upregulated in serum and tissue samples of GC patients [2]. As a recognized risk factor of GC, infection of *Helicobacter pylori* (*H. pylori*) contributes to the generation of ROS by cytotoxin-associated gene A (CagA) [6], subsequently activating Wnt, Ras, and mechanistic targets of rapamycin (mTOR) to drive the initiation of gastric carcinogenesis [7–10].

Increasing evidences indicate that oxidative stress is implicated in the pathological process of major depressive disorder (MDD). Similar with patients with GC, 8-OHdG increases in serum samples of patients with depression [11]. Low levels of antioxidant uric acid are associated with the occurrence of depression because of decreased levels of antioxidant properties [12]. The elevated levels of ROS are capable of decreasing the expression of brain-derived neurotrophic factor (BDNF), partially contributing to the progress of depression [13, 14]. With sparse whole-genome sequencing, Cai et al. identified sirtuin 1 (SIRT1) as a contributor to major depressive disorder [15]. SIRT1 functions by means of its antioxidant effects through enhancing antioxidant enzyme activity and inhibiting free radical-mediated oxidative injuries via decreasing nicotinamide adenine dinucleotide phosphate (NADPH) oxidase activation [16, 17]. All these suggest that oxidative stress plays crucial role in the process of depression.

With a high level of ROS, numerous oxidative stress-related proteins are dysregulated. SIRT1 and phosphoinositide 3-kinase (PI3K), for instance, are activated in both GC and depression [16, 18]. In our previous work, we found that ABL1 was upregulated in patients with both GC/colorectal carcinoma and depression [19, 20], and some kinds of cytokines like Leptin-LepRb and COX2 are abnormally expressed [21, 22]. Given the role of ROS in depression, GC, and crosstalk with inflammation, we speculated that ROS, ABL1, and inflammation are involved in the development of GC as well as GC-related depression. In this study, we designed series experiments to validate our hypothesis.

## 2. Patients and Methods

### 2.1. Patients

110 patients newly diagnosed with gastric adenocarcinomas were recruited in our study. They were diagnosed and received treatment from January 1, 2008 to December 31, 2016 in the Department of Oncology, the First Affiliated Hospital of Xi'an Jiaotong University. All the GC patients received examination and treatment following the National Comprehensive Cancer Network (NCCN) Clinical Practice Guidelines in Oncology. Follow-up was performed 3 times per year for the first 5 years, and annually thereafter. The project was terminated on December 31, 2016.

### 2.2. Instruments

During treatment and follow-up, assessment of the mood status among these patients was performed by physicians in the Department of Psychology, the First Affiliated Hospital of Xi'an Jiaotong University. The psychology assessment was performed every 6 months for 5 years and annually thereafter, and terminated when the patient died or when the study was terminated, which ever came earlier. We measured psychiatric symptoms with the method described in our previous work [23].

### 2.3. Ethical Standards

This study was approved by the First Affiliated Hospital of Xi'an Jiaotong University Ethics and Scientific Committee and met international standards for informed consent. All procedures followed were in accordance with the ethical standards of the responsible committee on human experimentation (institutional and national) and with the Helsinki Declaration of 1964 and later versions. Informed consent to be included in the study, or the equivalent, was obtained from all patients. All animal experiments were approved by the review board of Zhongnan Hospital of Wuhan University, and all institutional and national guidelines for the care and use of laboratory animals were followed.

### 2.4. Cell Culture

GC cell lines SGC-7901 and AGS were cultured in RPMI-1640 (HyClone) medium containing 10% fetal bovine serum (HyClone) and 1% penicillin/streptomycin (HyClone) in a 37°C incubator with a constant temperature and high humidity under 5% CO_2_ atmosphere.

### 2.5. MTT Assay

The MTT assay was used for evaluating cell proliferation. Briefly, cells in log phase were trypsinized into a single cell. Then, the GC cell lines were seeded into a 96-well plate (1 × 10^4^ cells/well) and cultured in 200 *μ*l of culture medium. 72 h later, the MTT solution (Sigma-Aldrich) (1 : 10 to complete culture medium) was added into each well. After incubation for 4 h, the supernatant of each well was aspirated carefully, and the crystals were dissolved with 100 *μ*l of DMSO (Sigma-Aldrich) by shaking for 5 min. The absorbance of each well was read by a microplate reader. The study was performed in triplicate.

### 2.6. Migration Assay

A wound-healing assay was used to detect cell migration. Briefly, the proper density of cells was passaged into a 6-well plate to ensure the 90%–100% monolayer cell confluence on the next day. Then, a 200 *μ*l pipette tip was used to form a wound. 48 hours later, the number of cells that migrated into the wound were recorded.

### 2.7. Drug Treatment

GC cell lines SGC-7901 and AGS were seeded into 96-well plates and treated with a serial concentration of Imatinib and Nilotinib (a gift from Novartis) to determine the half-maximal inhibitory concentration (IC50). Then, GC cells were treated with the two drugs at a dose of IC50 to explore the effect of ABL1 on inflammation signaling.

### 2.8. Flow Cytometric Analysis

Flow cytometry was accomplished with a FACS flow cytometer (BD Biosciences). SGC-7901 cells were stained with anti-E-cadherin (rabbit, Cell Signaling Technology) and anti-N-cadherin (mouse, Abcam) for 45 min, then stained with Alexa Fluor 488- and Alexa Fluor 594-labeled secondary antibody for 30 min after washing with PBS. All procedures were performed on ice. The analysis for the data was done with the FlowJo software (TreeStar Inc., Ashland, OR, USA).

### 2.9. PDX Model

The generation of the PDX mice model was established as described before [24]. Briefly, fresh surgical PDX tissue fragments (from GC patients of the Zhongnan Hospital of Wuhan University) (approximately 15 mm^3^) were implanted subcutaneously directly into male nude (8-week-old) mice (Hubei Provincial Laboratory Animal Public Service Center, Wuhan, China) and serial transplantation was conducted. The animal experiments in this study were performed according to the policies approved by IACUC. Tumor size was monitored twice per week, and tumor volumes (*V*) were calculated with the following formula: *V* = (length × (width)^2^)/2.

### 2.10. CMS

Mice were randomly exposed to several stressors in the morning and in the afternoon. The stressors included dampened sawdust, removal of sawdust, replacement of sawdust with water at 21°C, repeated alteration of sawdust, inclining the cage with 45°C, restraint stress, change of the dark and light cycle, and predator voice [25].

### 2.11. Behavioral Tests

#### 2.11.1. Open-Field Test (OFT)

OFT was adopted for measuring the spontaneous locomotor activities of mice. Briefly, mice were carefully placed in an open-field apparatus (50 cm × 50 cm) (Chengdu Techman Software Co. Ltd., Chengdu, China). The movement traces (5 min) of the mice were monitored by infrared cameras and analyzed with corresponding software [26].

#### 2.11.2. Sucrose Preference Test (SPT)

As described before, both water and food were denuded 24 h before SPT. During the test, two bottles with the same size were supplied to each animal: one with clean water and the other one with 1% sucrose solution. After 1 h, fluid consumptions were checked. Sucrose preference proportion was calculated with the following formula: sucrose solution consumption/(sucrose solution consumption + water consumption) × 100% [27].

#### 2.11.3. Tail Suspension Test (TST)

TST was performed for 6 minutes by suspending a mouse 30 cm from the floor. The immobile duration was monitored for a 6 min period. Mice were defined immobile only when they hung passively and were totally motionless [28].

### 2.12. Enzyme-Linked Immunosorbent Assay (ELISA)

The levels of 8-OHdG in patients' or mice' blood samples were quantitatively assayed with a DNA-damage ELISA kit (Ann Arbor, MI, USA). A hydrogen peroxide assay kit and a catalase assay kit were used to detect the levels of hydrogen peroxide and catalase in patients' blood samples (Nanjing Jiancheng Biotechnology Co., Nanjing, China). The concentrations of IL-6, IL-1*β*, COX2, and 5-HT of mice' blood samples were determined with a commercial ELISA kit for mouse IL-6, IL-1*β*, COX2, and 5-HT (Elabscience Biotechnology Co. Ltd., China). Procedures were performed based on the manufacturers' protocols, and the absorbance values were read at the recommended wave lengths.

### 2.13. Western Blot Analysis

Proteins from both the hippocampus and tumor tissue samples and cell lines were extracted. The concentration of proteins was detected with the BCA protein assay reagent (Shenzhen Wolsen Technology Co. Ltd., China). A total 15 *μ*g of proteins from each sample was loaded on 12% gel, then the proteins were transferred from the gel to the polyvinylidene difluoride membranes (EMD Millipore Corporation, Billerica, MA, USA), followed by blocking the membrane with 5% nonfat milk at room temperature for 2 h. The membrane was then incubated with a primary antibody specific for ABL1/p-ABL1(y412), IL-6, IL-1*β*, and COX2 (Abcam, Shanghai, China); NF-*κ*B1/p-NF-*κ*B1 (ser933) (Cell Signaling Technology, Danvers, MA, USA); STAT3/p-STAT3 (Tyr 705) (Santa Cruz Biotechnology (Shanghai) Co. Ltd., Shanghai, China); and *β*-actin antibody (Proteintech Group, Wuhan, China) at 4°C for 12 h. The membranes were washed 5 times with TBST (6 min each time), followed by 1 h incubation with the secondary antibody at room temperature. Image Lab software was used for the quantification of protein levels. Western blots were repeated three times for each sample.

### 2.14. Measurement of Serum Levels of ROS

The sera of mice were harvested, and serum levels of ROS were determined by the OxiSelect™ In Vitro ROS/RNS Assay Kit (Cell Biolabs Inc., California, USA) in accordance with the protocol provided. The fluorescence was read with a fluorescence plate reader at excitation 480 nm and emission 530 nm.

### 2.15. Statistical Analyses

Statistical analysis was performed by GraphPad Prism. Data are presented as mean ± SD. Significant difference at each comparison point is shown as ^∗^ *P* < 0.05 and ^∗∗^ *P* < 0.01.

## 3. Results

### 3.1. Depression Is Associated with Poor Prognosis of Patients with GC

The baseline characteristics of GC patients were presented in the supplementary materials (Supplementary Table 1). The prevalence of MDD was 38.18% (42/110) among the investigated patients with GC. Based on the OS of patients with GC, the cutoff of pLN (positive lymph node), NLR (neutrophil lymphocyte ratio), and LMR (lymphocyte monocyte ratio) were determined by a receiver operating characteristic (ROC) curve (Supplementary Table 2). Univariate Cox regression analysis indicated that pLN, TNM, and depression were associated with OS of patients with GC (Supplementary Table 3). TNM, pLN, and MDD were independent prognostic factors of patients with GC identified by multivariate analysis (Supplementary Table 4).

**Figure 1:**
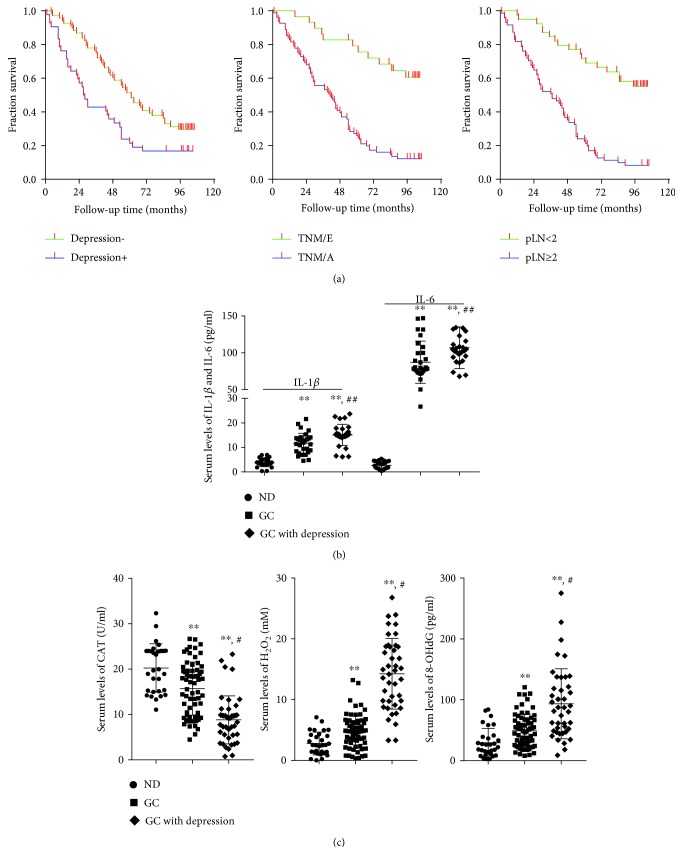
GC patients with depression experienced shorter OS and had dysregulated inflammation and oxidative stress markers. (a) The OS curves of patients generated with the Kaplan-Meier survival analysis indicated that GC patients with depression, advanced stage TNM, and pLN ≥ 2 experienced poorer OS. (b) Serum levels of inflammation markers significantly increased in GC patients with depression. (c) Oxidative stress makers significantly increased in GC patients with depression.

The median survival time of GC patients without or with depression was 61 months (95% CI, 48.18 to 73.82) and 27 months (95% CI, 20.65 to 33.65), *P* = 0.011; the median OS of the GC patients with early TNM was not reached (greater than the longest censoring time), and for the patients with advanced TNM, median OS was 41 months (95% CI, 28.97% to 53.03), *P* = 0.045; the median OS of the GC patients with pLN < 2 was not reached (greater than the longest censoring time), and the median OS of the patients with pLN ≥ 2 was 37 months (95% CI, 23.25 to 50.75), *P* = 0.002 (Figure 1(a)).

### 3.2. GC Patients with Depression Have Higher Levels of ROS, Accompanied with Dysregulated Oxidative Stress-Related Genes and Inflammation Factors

We previously confirmed that levels of ROS increased in patients with GC. Here, we tried to figure out the alteration of ROS in GC patients with depression. By ELISA assay, we found that serum levels of H_2_O_2_ and 8-OHdG dramatically increased, and serum levels of CAT significantly decreased in GC patients with depression (Figure 1(c)). With a quantitative real-time PCR array, we identified several oxidative stress-related genes differentially expressed, and ABL1 was significantly elevated in both GC patients with depression and colorectal carcinoma patients with depression [19, 20]. Inflammatory factors were reported to be associated with depression; as such, we tested the expression of IL-1*β* and IL-6 in serum samples of 30 normal donors (ND), 30 GC patients, and 30 GC patients with depression. It indicated that serum levels of IL-1*β* and IL-6 significantly increased in GC patients with depression (Figure 1(b)).

Moreover, as inflammation markers, high levels of NLR were significantly positively related with advanced TNM, pLN, and occurrence of depression. Conversely, high levels of LMR were significantly negatively associated with advanced TNM, pLN, and occurrence of depression (Supplementary Table 5). Evidences here suggested that inflammation might be involved in the development of GC and GC-related MDD.

### 3.3. ABL1 Is Correlated with the Inflammation Signaling Pathway in Patients with GC, and High Levels of ABL1 and STAT3 Were Associated with Poor OS of GC Patients

By retrieving the mRNA expression file on GC from The Cancer Genome Atlas (TCGA) data set (http://www.oncolnc.org), we discovered that the mRNA of ABL1 was positively associated with the mRNA of STAT3 and IL-6; the expression of STAT3 was positively correlated with the expression of NF-*κ*B1, NF-*κ*B2, IL-6, and IL-1*β*; the expression of NF-*κ*B1 was positively correlated with that of IL-6 and IL-1*β* (Supplementary Figure 1(a)).

Meanwhile, we analyzed the clinical data from the TCGA database to figure out the effect of ABL1, NF-*κ*B1, STAT3, IL-6, IL-1*β*, and COX2 on the OS of patients with GC. The result revealed that high levels of ABL1 and STAT3 were related to poor OS of GC patients (Supplementary Figure 1(b)).

### 3.4. ROS Regulate Activities of ABL1, Inflammation, Proliferation, Migration, and Epithelial-Mesenchymal Transition (EMT) of GC Cell Lines

**Figure 2:**
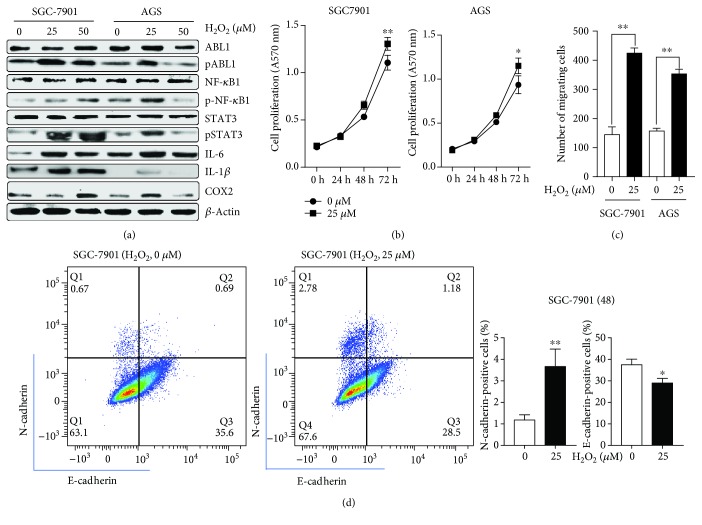
ROS regulated the activities of ABL1, inflammation, proliferation, migration, and EMT of GC cell lines. (a) H_2_O_2_ led to the alteration of ABL1 and inflammation factors in GC cell lines and EMT markers in GC cells. (b and c) H_2_O_2_ promoted the proliferation and migration of GC cells. (d) H_2_O_2_ induced the EMT of GC cells.

In our previous work, we summarized that ROS may regulate the expression of ABL1 by triggering receptor tyrosine kinases (RTKs), which subsequently led to the phosphorylation of ABL1. NF-*κ*B1 and STAT3 were the main factors involved in cytokine release [29, 30], and both NF-*κ*B1 and STAT3 were proven to be downstream targets of ABL1 in leukemia [31, 32]. Here, we treated SGC-7901 and AGS GC cell lines with different concentrations of H_2_O_2_ (0 *μ*M, 25 *μ*M, and 50 *μ*M), and the result showed that ROS could enhance the activities of ABL1 and could upregulate p-NF-*κ*B1, p-STAT3, IL-6, IL-1*β*, and COX2 (Figure 2(a)). The MTT assay and wound-healing assay showed that H_2_O_2_ could promote the proliferation and migration of GC cell lines (Figures 2(b) and 2(c)). The expression of E-cadherin and N-cadherin of SGC-7901 treated with H_2_O_2_ (25 *μ*M, 48 h) was analyzed by flow cytometry. The result indicated that the expression of N-cadherin significantly increased after H_2_O_2_ treatment, and the expression of E-cadherin significantly decreased (Figure 2(d)).

### 3.5. ABL1 Regulates Inflammatory Signaling Pathway

**Figure 3:**
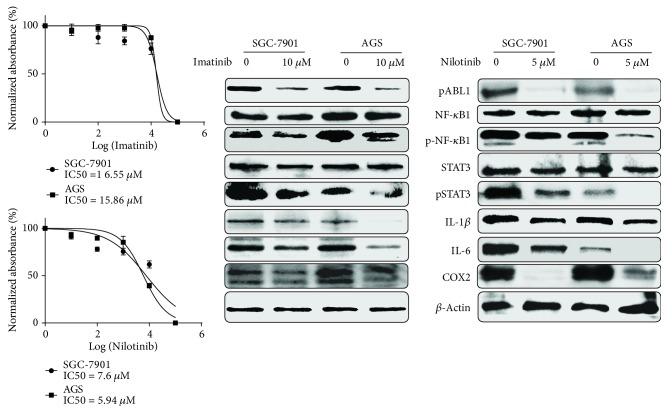
ABL1 regulated the inflammation signaling pathway. Blocking activities of ABL1 with Imatinib and Nilotinib inhibits the inflammatory signaling pathway including activities of NF-*κ*B1, STAT3, IL-1*β*, IL-6, and COX2.

To investigate the association of ABL1 and inflammation, we blocked the activities of ABL1 with its inhibitors Imatinib and Nilotinib. By treating GC cell lines SGC-7901 and AGS with Imatinib (10 *μ*M) and Nilotinib (5 *μ*M) for 24 h, the levels of pABL1 decreased, accompanied with the inhibition of p-NF-*κ*B1, p-STAT3, IL-6, IL-1*β*, and COX2, which meant that ABL1 regulated the inflammatory signaling pathway (Figure 3).

### 3.6. CMS Leads to Depressive-Like Behaviors and Development of GC

To better understand the relationship of ROS, cytokine release, development of cancer, and cancer-related depression, we generated the CMS-induced mice model and the GC PDX model. During exposure to CMS for 4 weeks, depressive-like behaviors were monitored with OFT, SPT, and TST. Mice showed significantly less total move distance, longer immobile time, and lower sucrose preference and sucrose consumption induced with CMS (Supplementary Figure 2(a)). The ELISA assay indicated that serum levels of IL-6, IL-1*β*, COX2, ROS, and 8-OHdG significantly increased, and serum levels of 5-HT significantly decreased in CMS mice compared with the control (Supplementary Figure 2(b)).

**Figure 4:**
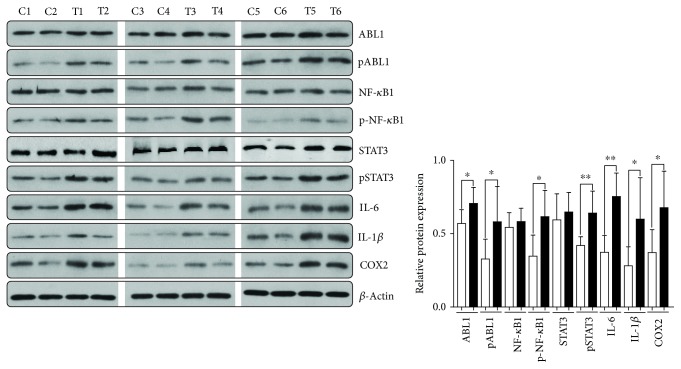
ABL1 and inflammation factors were dysregulated in the hippocampus tissue of CMS-induced mice (*n* = 6). ABL1, NF-*κ*B1, STAT3, IL-1*β*, IL-6, and COX2 were upregulated in the hippocampus tissue of CMS-induced mice. C, mice from the control group; T, mice from the CMS-treated group.

As shown in Figure 2, H_2_O_2_ could enhance the activities of ABL1, as well as increase the levels of p-NF-*κ*B1, p-STAT3, IL-6, IL-1*β*, and COX2. Consisting of H_2_O_2_-treated GC cell lines in vitro, ABL1, pABL1, p-NF-*κ*B1, p-STAT3, IL-6, IL-1*β*, and COX2 were significantly increased in the hippocampus tissue of CMS-induced mice (Figure 4).

**Figure 5:**
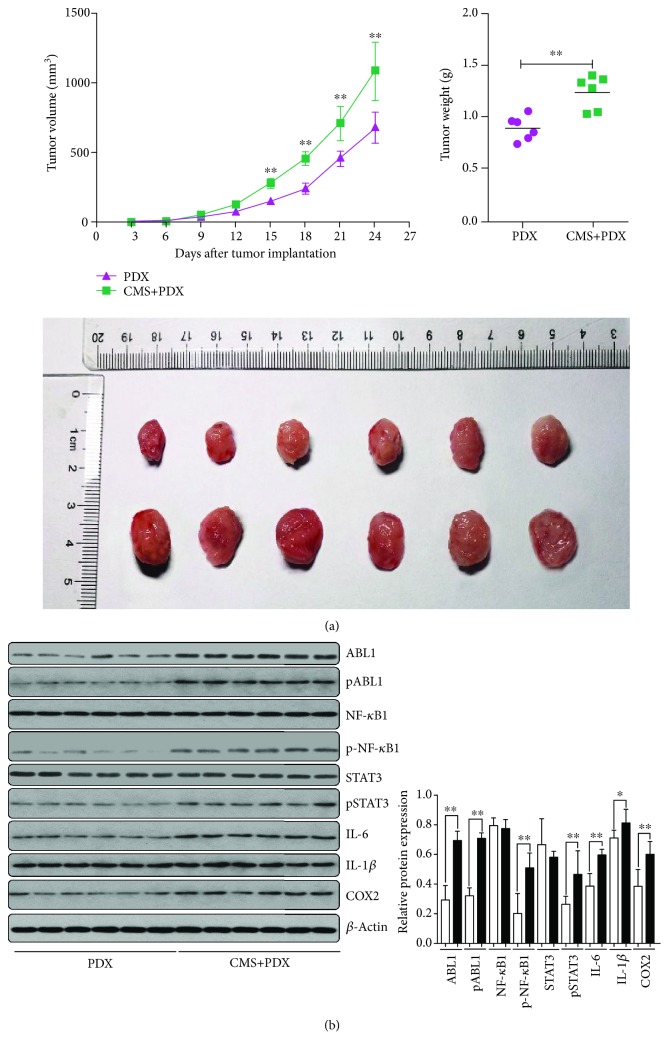
CMS facilitated the development of GC, accompanied with dysregulated activities of ABL1 and the inflammation signaling pathway. (a) CMS leads to the development of GC. Tumor growth curve and tumor weight indicated that CMS leads to the development of GC (*n* = 6). (b) ABL1 and inflammation factors including NF-*κ*B1, STAT3, IL-1*β*, IL-6, and COX2 were dysregulated in tumor tissues of CMS-induced PDX mice (*n* = 6).

To investigate the effect of CMS on GC, PDX mice models were established after 4 weeks of exposure to CMS. The results showed that CMS could promote tumor growth (Figure 5(a)). Similarly, in tumor tissues of CMS-induced mice, the expression and phosphorylation of ABL1, p-NF-*κ*B1, p-STAT3, IL-6, IL-1*β*, and COX2 significantly increased (Figure 5(b)).

### 3.7. Tumor Bearing Leads to Depressive-Like Behaviors of Mice

**Figure 6:**
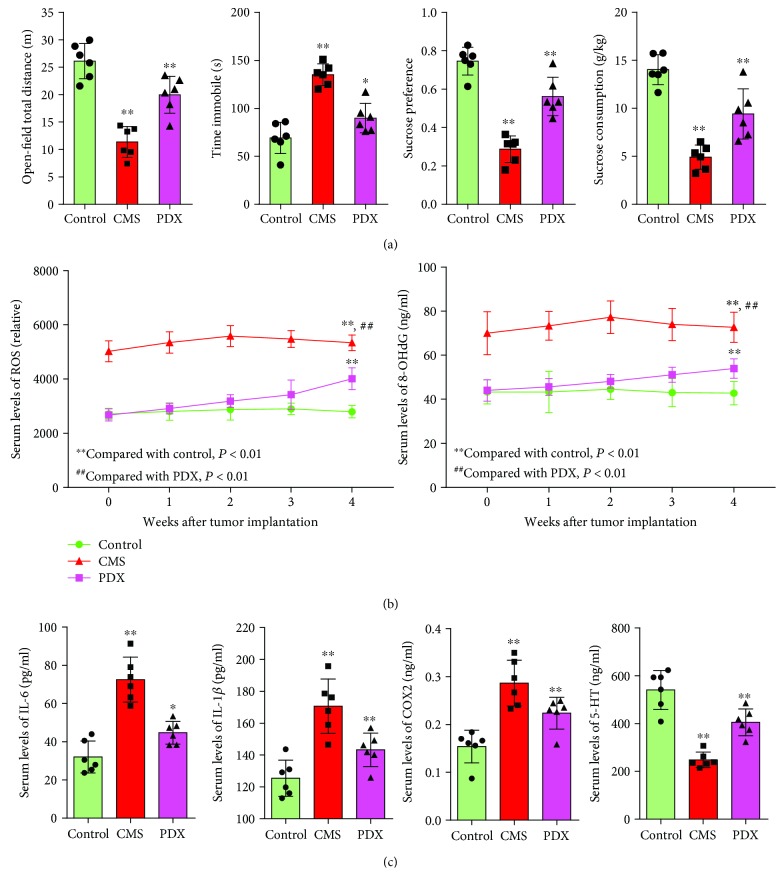
Tumor bearing led to depressive-like behaviors of mice and dynamic change of ROS and inflammation factors. (a) Tumor bearing led to depressive-like behaviors of mice. (b and c) Tumor bearing led to increased levels of ROS and dysregulated inflammation factors (*n* = 6).

To investigate the effect of tumor on depression, we constructed a PDX-bearing mice model. After 4 weeks of PDX bearing, mice showed significantly less total move distance, longer immobile time, and lower sucrose preference and sucrose consumption compared with the control (Figure 6(a)). Serum levels of ROS and 8-OHdG significantly increased in PDX-bearing mice (Figure 6(b)). IL-6, IL-1*β*, and COX2 significantly increased in PDX-bearing mice, while 5-HT significantly decreased in PDX-bearing mice (Figure 6(c)).

## 4. Discussion

Based on the clinical data we gathered from GC patients with depression, MDD is an independent factor predicting OS of GC. As such, we used the CMS-induced mice to probe the effect of depression on the development of GC. For mice treated with CMS, serum levels of ROS and cytokines (IL-6, IL-1*β*, and COX2) significantly increased; the tumor grew faster in CMS-induced mice. These results from the mice model were consistent with what we gathered from the clinical data of patients with GC. Moreover, after tumor bearing for 4 weeks, we observed depressive-like behaviors occurring among these mice, and the levels of ROS and 8-OHdG dynamically elevated after tumor bearing.

Our preliminary study confirmed that ABL1 was expressed notably higher in GC and colorectal carcinoma patients with depression. Encouragingly, with whole genome cRNA microarrays, Yi et al. gathered 30 differentially expressed genes in patients with MDD, including ABL1 [33]. In addition, ROS-mediated ABL1 plays key role in Parkinson's disease [34]. The dysregulation of ROS may influence the expression of ABL1 through triggering genomic instability [35]. In renal carcinoma, the levels of ROS are positively correlated with the expression of ABL1 [36]. H_2_O_2_, a type of ROS, promotes the expression of ABL1, and the ROS scavenger N-acetylcysteine (NAC) restrains the activities of ABL1. In both CMS-induced mice and PDX mice, exposure to CMS significantly increased ROS levels; meanwhile, both expression of ABL1 and phosphorylation of ABL1 were elevated in the hippocampus of CMS-induced mice and in the tumor tissue of PDX mice exposed to CMS. In an *in vitro* study, we treated GC lines with H_2_O_2_ and found that both ABL1 and phosphorylation of ABL1 were H_2_O_2_ dose dependent [37]. Collectively, these results indicated that ROS could trigger the activities of ABL1 in GC.

Studies reported that the ROS-mediated inflammatory microenvironment plays a crucial role in tumorigenesis and tumor metastasis. NF-*κ*B1 and STAT3 are crucial factors regulating the tumor microenvironment. Both NF-*κ*B and STAT3 are proven to be downstream targets of ABL1 in leukemia [31, 32]. The constitutive activation of NF-*κ*B1 leads to cytokine release (TNF*α*, IL-6, IL-1, and IL-8), and these cytokines can give a positive feedback loop to induce the activation of NF-*κ*B [38–40]. Besides, by interacting with PI3K-AKT-mTOR, NF-*κ*B controls tumor proliferation through the regulation of c-myc and cyclin D1 [41–43]. Similarly, there is also a positive feedback loop between cytokine release and the activation of STAT3, and STAT3 is required for tumor transformation [31]. Indeed, cytokines promote self-renewal and EMT of tumor cells, which may be crucial in treatment resistance and the relapse of cancer [44]. Zhu et al. found that IL-6 could upregulate the expression of CD44, a marker of cancer stem cells, in GC cells [45]; IL-1*β* may be involved in gastric dysplasia by regulating stromal cell-derived factor-1 [46]; and inhibition of COX2 could convert the preneoplastic lesions by the regulation of prostaglandin E-2 [47]. Taking both clinical and experimental data from in vivo and in vitro studies together, ROS mediate the activation of ABL1 to promote cytokine release by triggering NF-*κ*B and STAT3, which may partially account for the poorer prognosis of GC patients with depression.

To probe the potential mechanism of the occurrence of depression, we also detect the expression of ABL1, NF-*κ*B1, STAT3, IL-6, IL-1*β*, and COX2 in the hippocampus of depressive mice. Interestingly, ROS seems to activate ABL1/NF-*κ*B1-related cytokines (IL-6, IL-1*β*, and COX2), thus involving itself in the process of depression. These cytokines interact with the HPA axis, leading to the occurrence of depression [48, 49]. Actually, Norden et al. found that tumor growth could increase neuroinflammation, with increased IL-1*β* and IL-6 expression in the cortex and the hippocampus of tumor-bearing mice [50]. Some also think that ROS are the by-products of MDD-related dysfunctional mitochondria, leading the activation of inflammation-related signaling pathways including NF-*κ*B and NRF2, which in turn promotes the progression of MDD [51, 52]. For GC patients with depression, inflammation markers NLR and LMR were associated with depression. Furthermore, we also investigated the effect of tumor bearing on depression, and found that GC bearing can lead to depressive-like behaviors of mice. The levels of ROS, IL-6, IL-1*β*, and COX2 significantly increased at 4 weeks posttumor implantation. All these convinced us that ROS-ABL1-related inflammation factor release played a key role in the occurrence of depressive-like behavior among tumor-bearing mice.

## 5. Conclusion

ROS-activated ABL1 mediates the inflammatory signaling pathway, which subsequently leads to the development of GC and GC-related depression.

## Data Availability

The datasets used and analyzed during the current study are available from the corresponding author upon reasonable request.

## Supplementary Materials

Supplementary Materials Supplementary Table 1: the baseline characteristics of investigated GC patients. Supplementary Table 2: the cutoff of pLN, NLR, and LMR determined by ROC. Supplementary Table 3: univariate Cox regression analysis of factors associated with OS of GC patients. Supplementary Table 4: multivariate analysis of factors affecting OS of GC patients. Supplementary Table 5: correlation of NLR and LMR with clinical characteristics of GC. Supplementary Figure 1: (a) ABL1 is correlated with the inflammation signaling pathway in patients with GC (b) and high levels of ABL1 and STAT3 were associated with poor OS of GC patients. Supplementary Figure 2: (a) CMS led to depressive-like behaviors in mice. (b and c) CMS induced dysregulated inflammation factors and ROS.
