# Supplementary material for: Reactive Oxygen Species Are Involved in the Development of Gastric Cancer and Gastric Cancer-Related Depression through ABL1-Mediated Inflammation Signaling Pathway
Source: Oxid Med Cell Longev. 2019 Jul 15;2019:5813985. doi: 10.1155/2019/5813985 (PMC6664690; doi:10.1155/2019/5813985)
Supplement: Supplementary Materials — Supplementary Table 1: the baseline characteristics of investigated GC patients. Supplementary Table 2: the cutoff of pLN, NLR, and LMR determined by ROC. Supplementary Table 3: univariate Cox regression analysis of factors associated with OS of GC patients. Supplementary Table 4: multivariate analysis of factors affecting OS of GC patients. Supplementary Table 5: correlation of NLR and LMR with clinical characteristics of GC. Supplementary Figure 1: (a) ABL1 is correlated with the inflammation signaling pathway in patients with GC (b) and high levels of ABL1 and STAT3 were associated with poor OS of GC patients. Supplementary Figure 2: (a) CMS led to depressive-like behaviors in mice. (b and c) CMS induced dysregulated inflammation factors and ROS. [file 5813985.f1.pptx]

## Slide 1
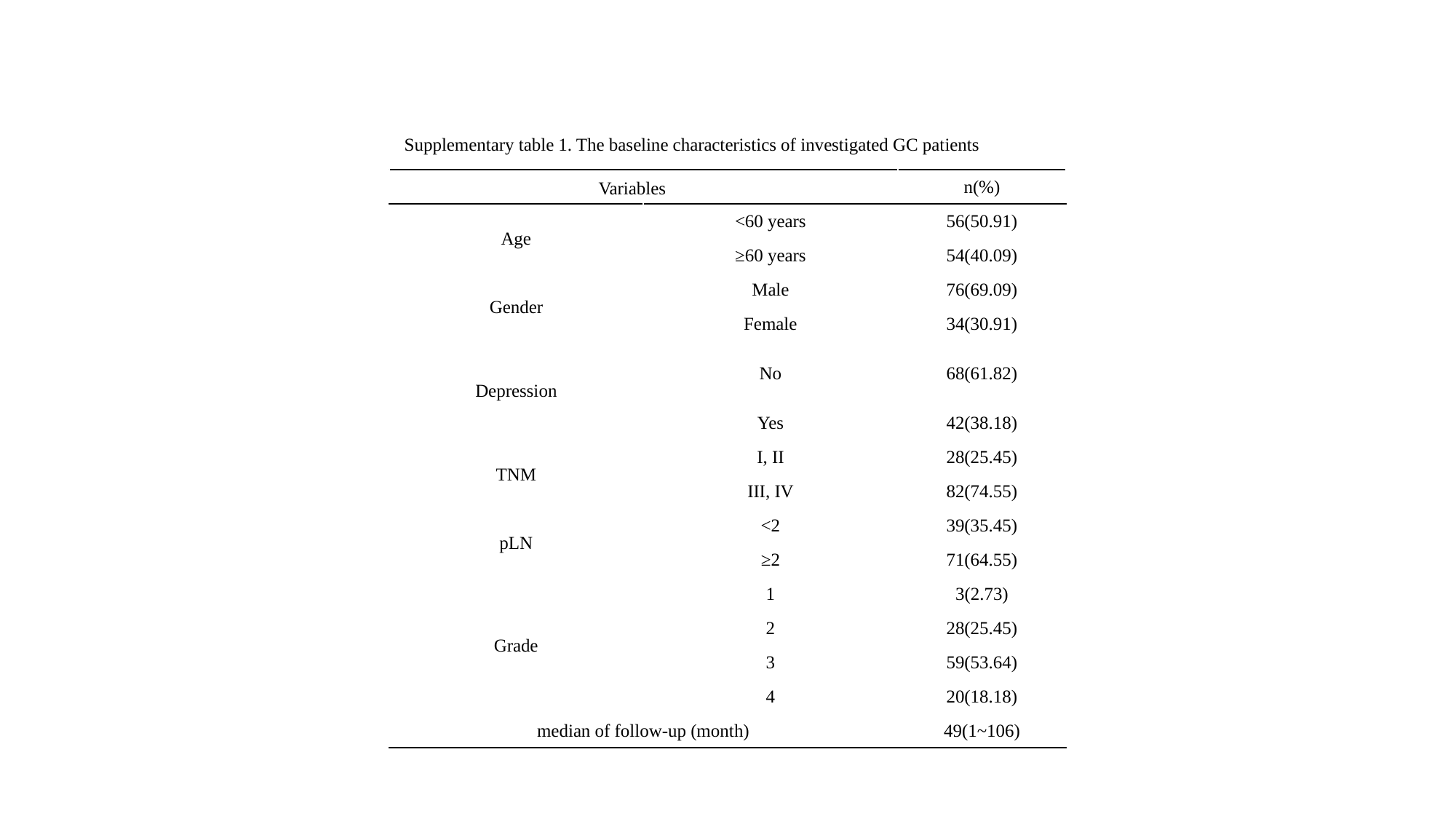

Supplementary table 1. The baseline characteristics of investigated GC patients
| Variables | | n(%) |
| --- | --- | --- |
| Age | <60 years | 56(50.91) |
| | ≥60 years | 54(40.09) |
| Gender | Male | 76(69.09) |
| | Female | 34(30.91) |
| Depression | No | 68(61.82) |
| | Yes | 42(38.18) |
| TNM | I, II | 28(25.45) |
| | III, IV | 82(74.55) |
| pLN | <2 | 39(35.45) |
| | ≥2 | 71(64.55) |
| Grade | 1 | 3(2.73) |
| | 2 | 28(25.45) |
| | 3 | 59(53.64) |
| | 4 | 20(18.18) |
| median of follow-up (month) | | 49(1~106) |

## Slide 2
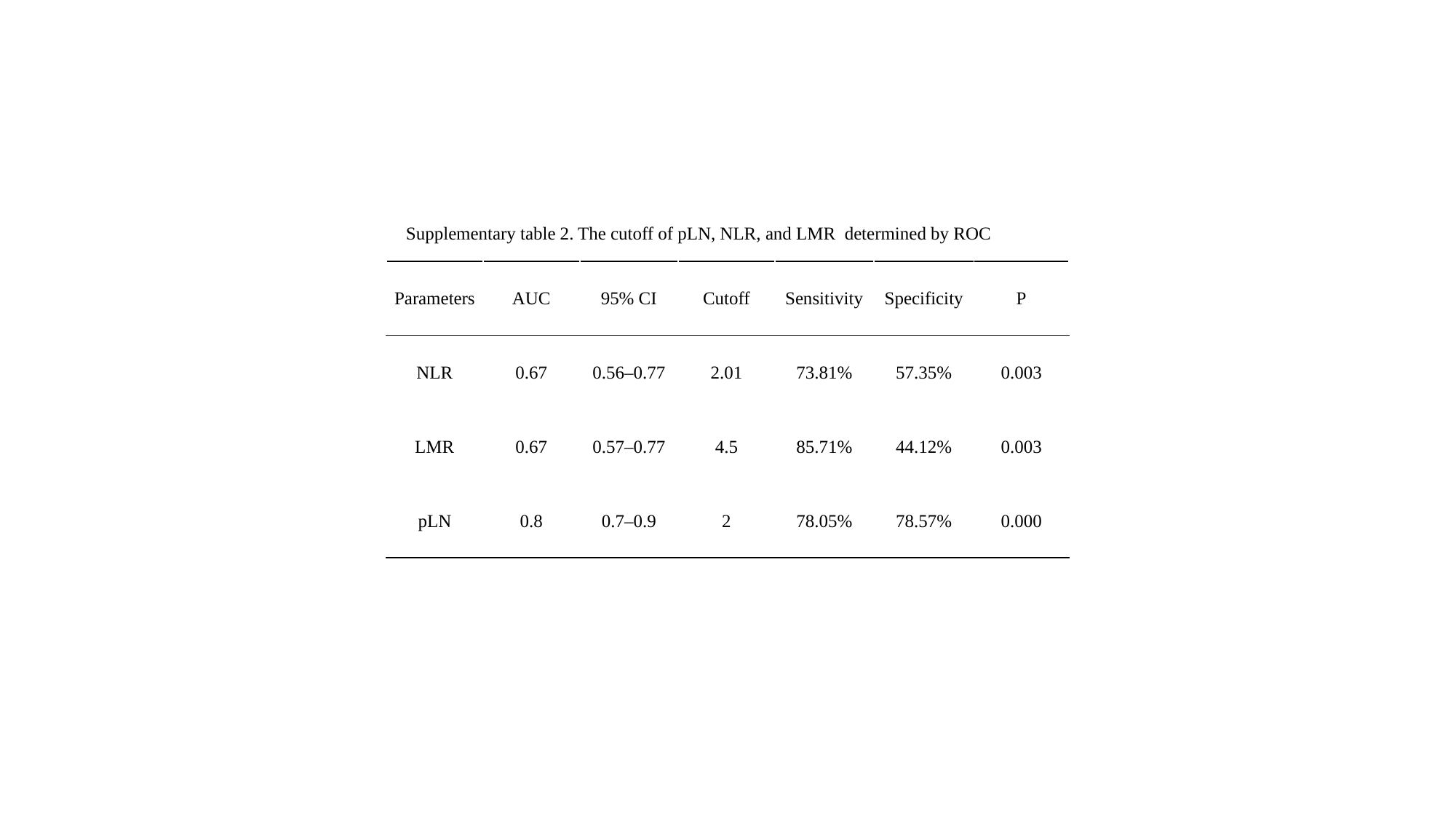

Supplementary table 2. The cutoff of pLN, NLR, and LMR determined by ROC
| Parameters | AUC | 95% CI | Cutoff | Sensitivity | Specificity | P |
| --- | --- | --- | --- | --- | --- | --- |
| NLR | 0.67 | 0.56–0.77 | 2.01 | 73.81% | 57.35% | 0.003 |
| LMR | 0.67 | 0.57–0.77 | 4.5 | 85.71% | 44.12% | 0.003 |
| pLN | 0.8 | 0.7–0.9 | 2 | 78.05% | 78.57% | 0.000 |

## Slide 3
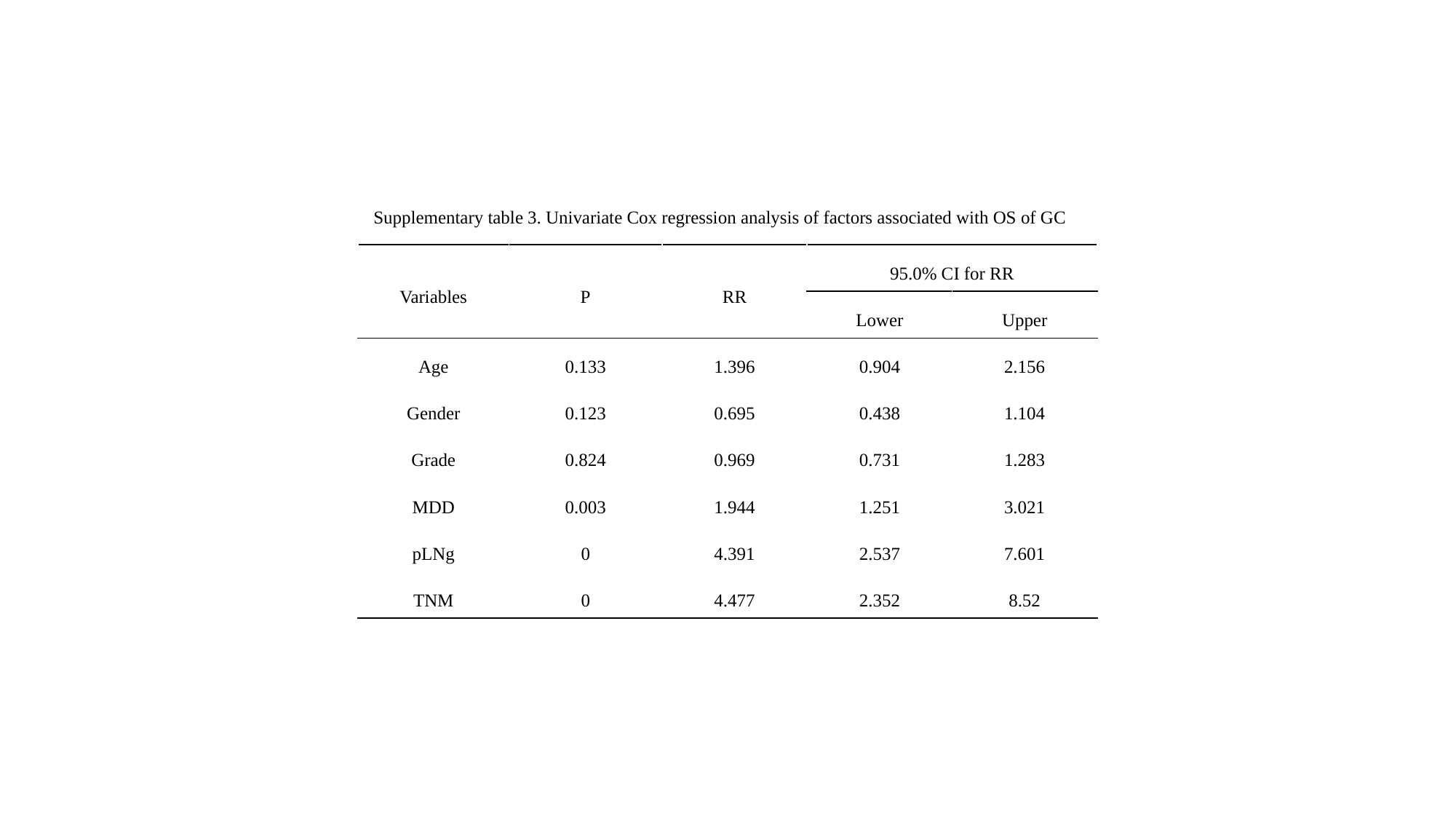

Supplementary table 3. Univariate Cox regression analysis of factors associated with OS of GC
| Variables | P | RR | 95.0% CI for RR | |
| --- | --- | --- | --- | --- |
| | | | Lower | Upper |
| Age | 0.133 | 1.396 | 0.904 | 2.156 |
| Gender | 0.123 | 0.695 | 0.438 | 1.104 |
| Grade | 0.824 | 0.969 | 0.731 | 1.283 |
| MDD | 0.003 | 1.944 | 1.251 | 3.021 |
| pLNg | 0 | 4.391 | 2.537 | 7.601 |
| TNM | 0 | 4.477 | 2.352 | 8.52 |

## Slide 4
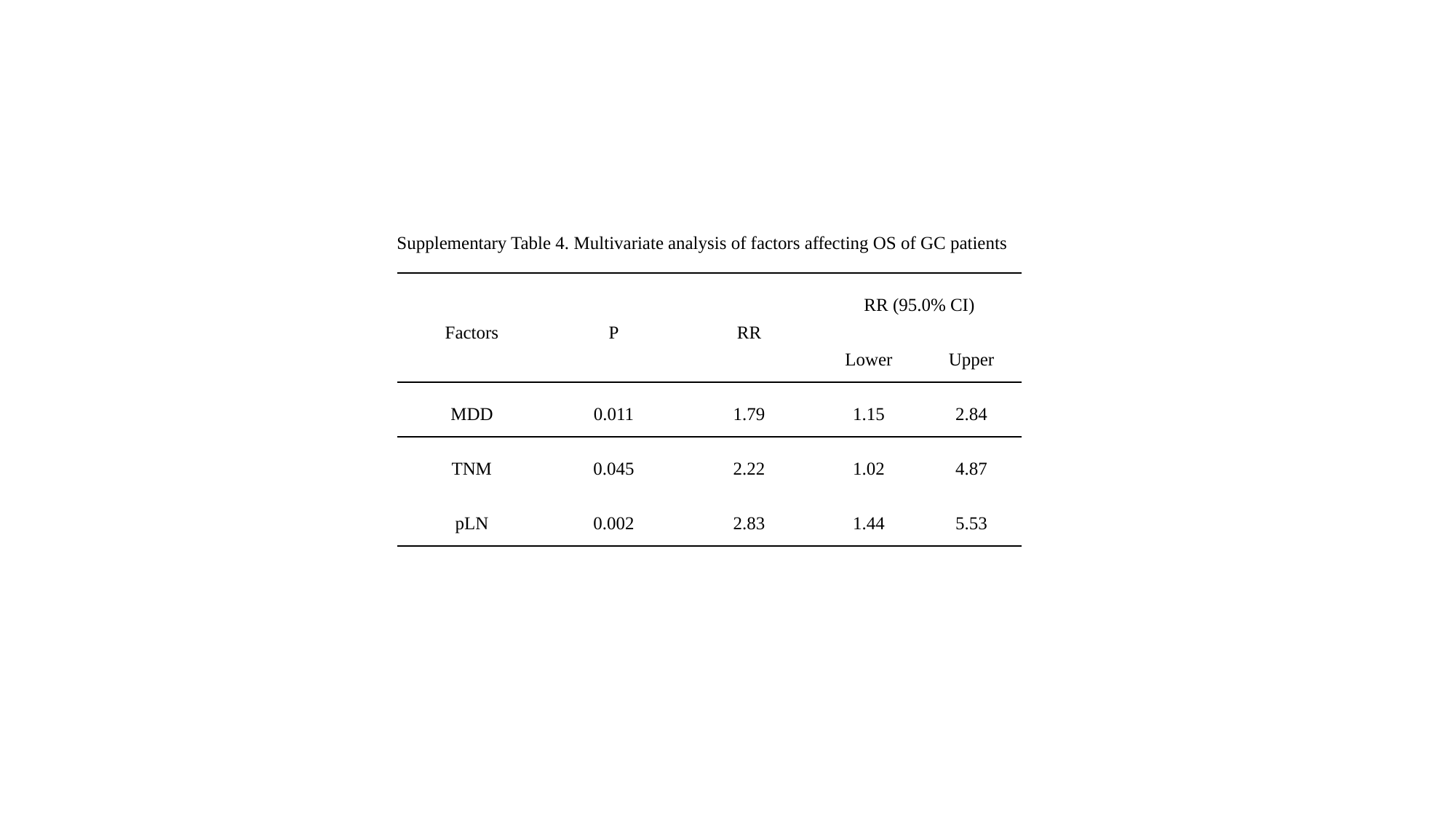

Supplementary Table 4. Multivariate analysis of factors affecting OS of GC patients
| Factors | P | RR | RR (95.0% CI) | |
| --- | --- | --- | --- | --- |
| | | | Lower | Upper |
| MDD | 0.011 | 1.79 | 1.15 | 2.84 |
| TNM | 0.045 | 2.22 | 1.02 | 4.87 |
| pLN | 0.002 | 2.83 | 1.44 | 5.53 |

## Slide 5
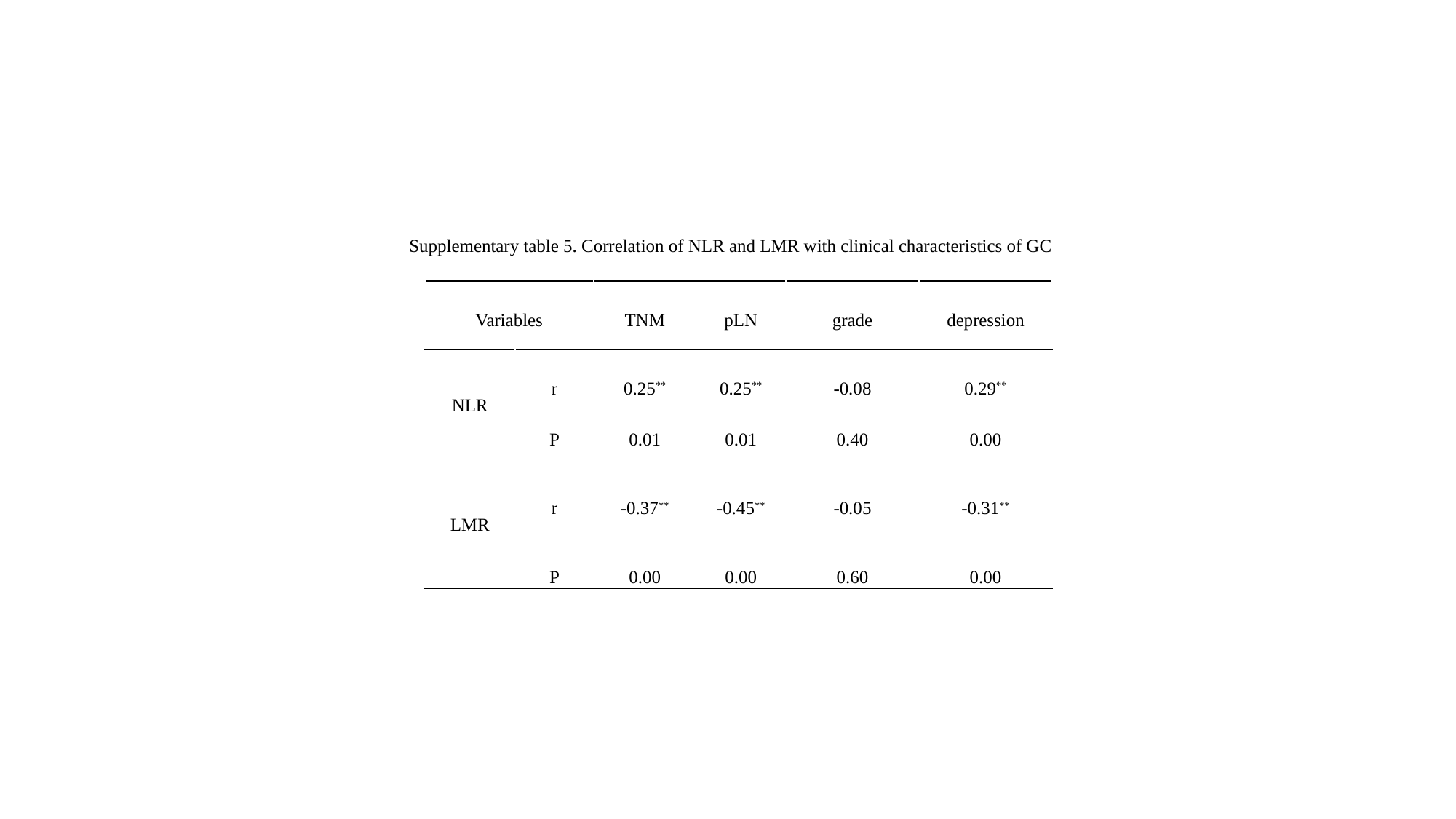

Supplementary table 5. Correlation of NLR and LMR with clinical characteristics of GC
| Variables | | TNM | pLN | grade | depression |
| --- | --- | --- | --- | --- | --- |
| NLR | r | 0.25\*\* | 0.25\*\* | -0.08 | 0.29\*\* |
| | P | 0.01 | 0.01 | 0.40 | 0.00 |
| LMR | r | -0.37\*\* | -0.45\*\* | -0.05 | -0.31\*\* |
| | P | 0.00 | 0.00 | 0.60 | 0.00 |

## Slide 6
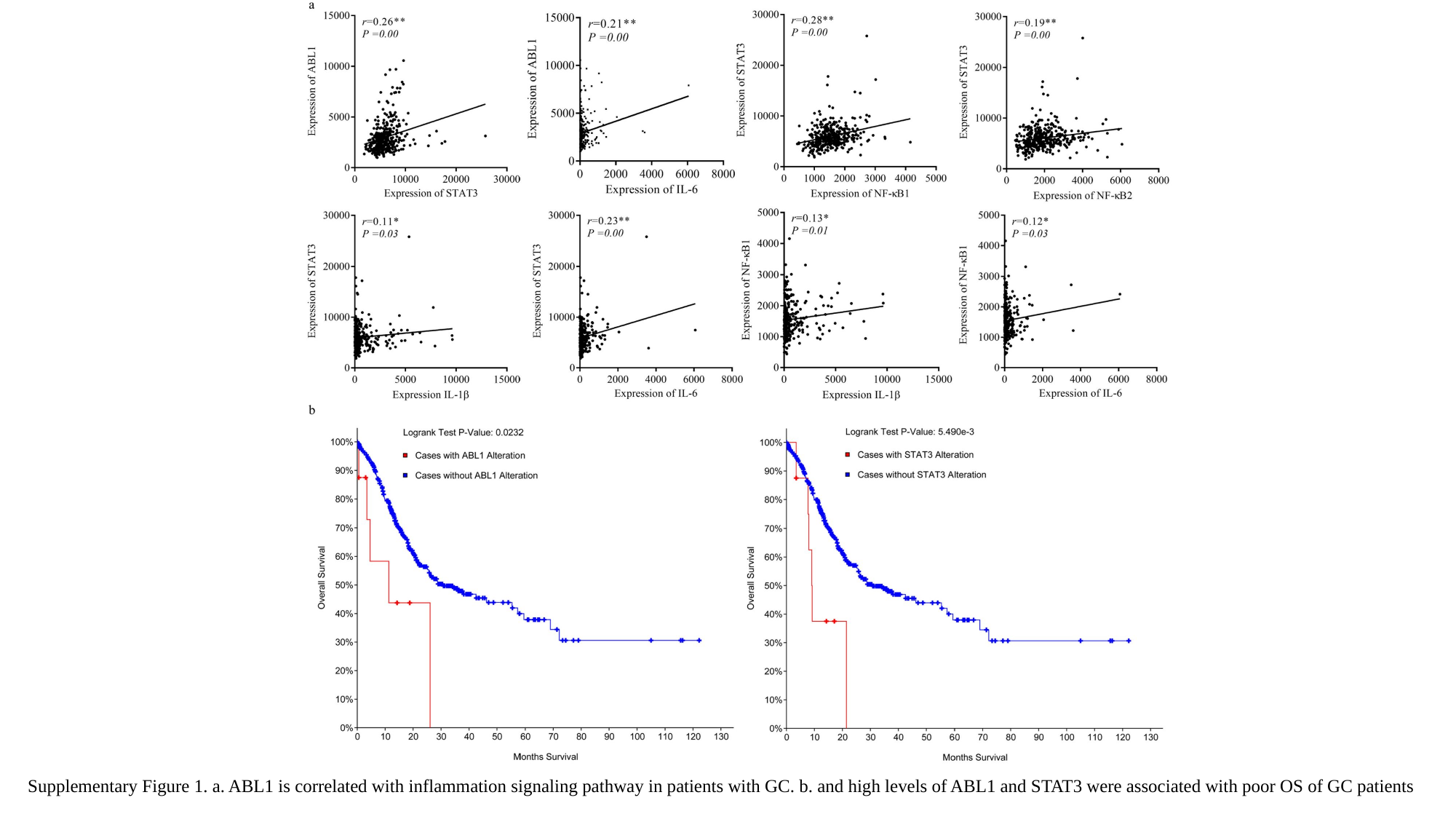

Supplementary Figure 1. a. ABL1 is correlated with inflammation signaling pathway in patients with GC. b. and high levels of ABL1 and STAT3 were associated with poor OS of GC patients

## Slide 7
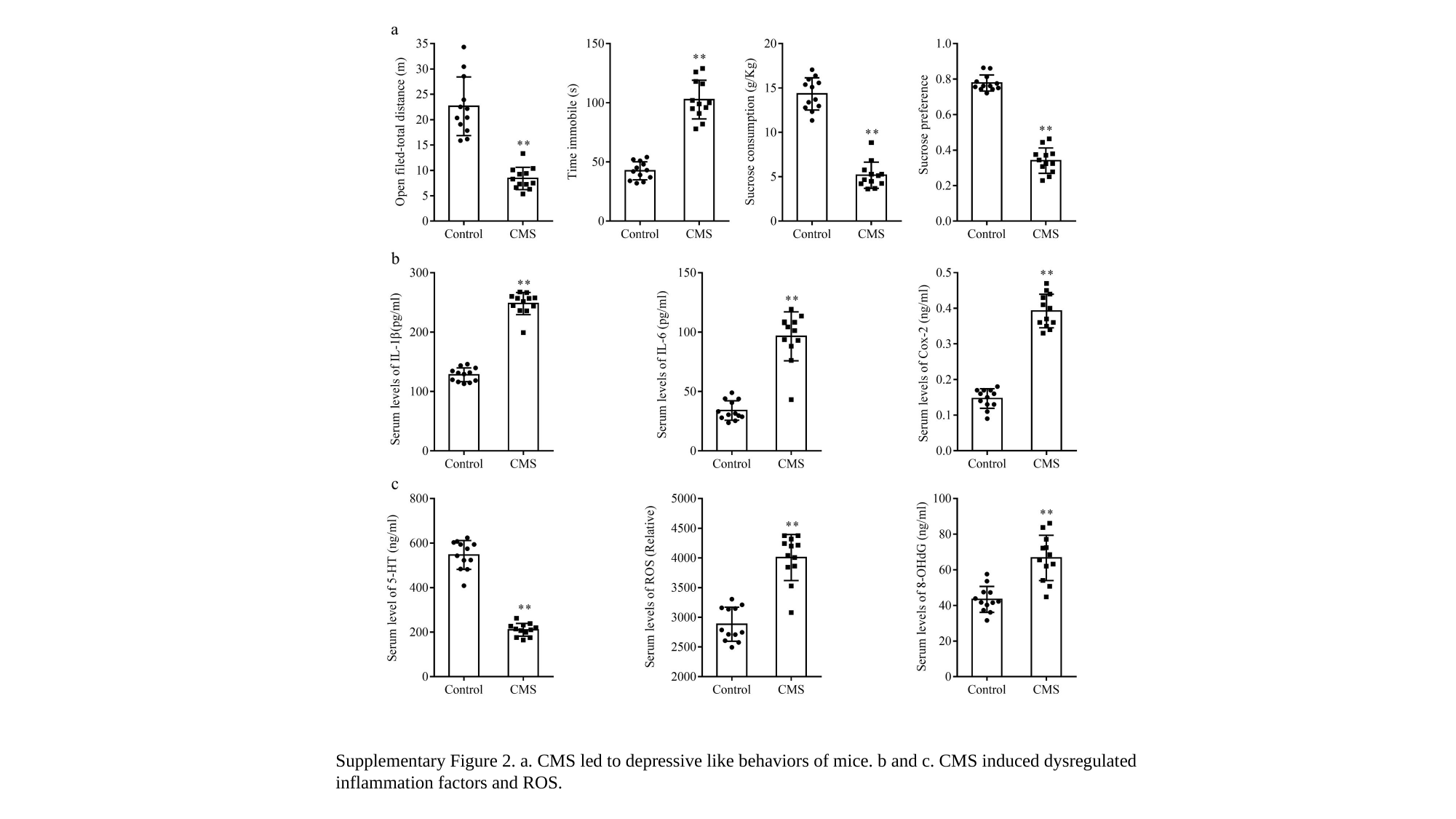

Supplementary Figure 2. a. CMS led to depressive like behaviors of mice. b and c. CMS induced dysregulated inflammation factors and ROS.
